# Fictional biomedical bibliographies in the Era of ChatGPT: Don´t revive Dr. O. Úplavici (1887–1938)

**DOI:** 10.1007/s00428-025-04100-x

**Published:** 2025-04-15

**Authors:** Abbas Agaimy

**Affiliations:** Institute of Pathology, Friedrich-Alexander-University Erlangen-Nürnberg, University Hospital Erlangen, Krankenhausstrasse 8 - 10, 91054 Erlangen, Germany

Appropriate citation is the mainstay of scientific continuity, integrity, and honesty in biomedical research. The major goal of citing prior works is to provide readers/researchers with a valid and integral account of what has been done, at same time acknowledging the primacy of prior works on the topic. However, the process of appropriate citation (completeness & correctness, appropriate context quoting, credit giving to others, etc.) not rarely is complicated by several technical, cultural, and behavioral factors that may negatively impact correct quotation of the literature. Technical factors mainly relate to inappropriate literature search (cursory/incomplete literature review, failure to explore secondary literature, language barriers, limited access to full articles, and limited familiarity with the research topic). In a trial to obtain credit or primacy, authors might intentionally ignore or inappropriately cite prior works.

In this issue of VIAR, Guastafierro et al. (10.1007/s00428-024-03918-1) evaluated the use of ChatGPT, an AI system capable of processing and generating human-like language, in addressing pathology-related diagnostic questions and its ability to provide scientific references [1]. Remarkably, 30% of bibliographic references generated by ChatGPT were incorrect (12.1% inaccurate and 17.8% non-existing) [1]. The authors concluded that ChatGPT-generated imprecise referencing highlights the risk of using it as a self-learning tool and underscores the irreplaceable role of human experts in synthesizing and compiling clinical data into concise meaningful reports [1]. In the context of Guastafierro et al.´s study, the Editor-in-Chief would like to draw attention of the readers to an unpreceded and largely unknown historical mis-citation that, inadvertently, had created a fictitious scientist named “Dr. O. úplavici” in 1887.

## The biography of the fictitious Dr. O. úplavici (1887–1938) and tribute to Jaroslav Hlava (I855–1924), the real scientist hiding behind “Dr. O. úplavici “

Professor Jaroslav Hlava, graduated from the Medical Faculty of Charles University in Prague in 1879. In 1887, he became a full professor of pathological anatomy, thereafter rector of Prague's General Hospital and director of the Czech Institute of Pathological Anatomy. In addition to his board interest in the science of diseases, Professor Hlava was a pioneer in bacteriology and the studies of etiology of infectious diseases. In 1887, he authored a widely cited seminal article entitled "O uplavici; Predbezne sdeleni" ("On dysentery; a preliminary communication."), in which he illustrated for the first time that diarrhoea caused by *Entamoeba histolytica* is transmittable from man to cats by intrarectal inoculation of infected fecal material from the former into the latter. Professor Hlava's paper (written in the Bohemian/Czech language) was published in the leading Czech medical journal of that time [2].

## Mis-citation and the birth of Dr. O Uplavici in (1887–1938)

Since its original publication, Hlava´s paper has not been translated or published into any form of scientific communication in any other language, except for a brief German language abstracting published by Dr Kartulis in the journal “Zentralblatt für Bakteriologie und Parasitenkunde in same year (vol. I, P. 537; I887). However, during the translation, the author's name (Hlava; written in small characters) became entirely omitted, and, instead, the first part of the title “*O ÚPLAVICI”* (“*About dysentery”*) was mistaken for the name of the author ("Uplavici, O."), while the subtitle “Předběžné Sdělení,"(= preliminary communication “) replaced the full title, resulting in the very first appearance of “0. Uplavici of Prague” in the field of amoebic dysentery research. Indeed, it was not only this mis-cite of the author´s name, but also the personal referral of the translator, Dr. Kartulis, to his own correspondence with Dr. Uplavici, that enhanced and declared the definitive birth of the new doctor, "Uplavici, O". This translation error might have been influenced by misinterpretation of the Czech name “Hlava” which also means 'Title or head'. This dramatic oversight of the original authors´ name gave credit for this novel medical discovery to "Uplavici, 0"(Dysentery, On).' As a consequence, the article became universally attributed to O. Uplavici in subsequent English publications as investigators and reviewers around the globe had referred to the classic experiments on amoebic dysentery by Dr. O. Uplavici in respectful books and periodicals for the next 50 years [3].

## The sudden death of Dr. Uplavici, O.

Like many other workers, the English protozoologist Dr. Clifford Dobell of London initially knew of Dr. Uplavici´s work only by hearsay. However, his scrutiny to assign Dr. Uplavici properly in the bibliography of a monograph he was preparing on dysentery in animals, ultimately uncovered the truth of the mythical worker Dr. “Uplavici, O”. After a long, exhaustive search for the full original article of Dr. Hlava, Dr. Dobell´s effort was successful in obtaining a complete typewritten copy of Hlava's original paper from Dr. J. Drbohlav of Prague in 1924, that was then translated into English by Dr. F. Simer of Bratislava. It was these three workers, whom the scientific community should be much thankful in finally clarifying the true story of Dr. Uplavici.

After a 50-years of flourishing scientific life in bacteriology and parasitology, ranking him with the giants in the field such as Pasteur, Koch, and Lister, the scientific and the fictitious biological life of the mythical bacteriologist "Dr. 0. Uplavici" ended acutely when the mistake was discovered and corrected by Dobell in 1938 after reading the whole translated original paper, who then became much lucky to write the formal obituary of "Dr. 0. Uplavici"(1887–1938)". In his obituary, Dr. Dobell summarized the life of "Dr. 0. Uplavici" as “a pure Czech with a Greek father and a German mother, born in I887, published his only paper in the same year, obtained his doctor's degree later in the United States, and then breathed his last in England” [4–6].

## Lessons learned from the story of Dr. O. UPLAVICI

Failure to retrieve and read the original publication before citing it (authors frequently cite papers only through reading about them in other papers or extracting them from the secondary literature in a sense of “knowledge by hearsays”) would ultimately result in inappropriate citations. Indeed, it was not only those cursory writers who followed and reproduced the mis-citation of "0. Uplavici", but also the well-known Index Catalogue of Medical and Veterinary Zoology (compiled by Stiles & Hassal, Washington), that has not only cited Uplavici by name, but also offered him for the first time his degree (“Dr.”) [4–6].

While the creation of the mythical "Dr. 0. Uplavici" has had little harmful consequences for the field of bacteriology at that time, it wouldn´t have been the same harmless effect in our time, given the tremendous relevance of citation records for the ranking of individual medical and research professionals and for journal citation records. Indeed, attributing citations to the false or to a fictitious author/s has enormous negative impact for the true author/s and journal/s. Moreover, creating fictional bibliographic sources would have been used and abused for creating erroneous study designs and hypotheses that would, at least in short term, have resulted in disastrous funding and ranking mishandles. This is particularly true in case the AI-based tools be used for self-learning or for self-evaluation [7]. Accordingly, these emerging tools pose a great challenge to biomedical journals when handling manuscripts for potential publication. It cannot be overemphasized that efficient digital tools and programmes are urgently needed to be established and implemented by biomedical journals to verify appropriate bibliographic citations as well as verifying manuscript integrity. Last but not least, the academic/scientific, medicolegal, and ethical clearance for the increasing use of AI-based algorithms for creating scientific hypotheses, writing articles, generating bibliographic data, processing grant applications and being involved in manuscript evaluation (as potential substitutes for formal peer review) have to be addressed by responsible authorities and institutions in a satisfactory manner.

The illustrated "Dr. 0. Uplavici" biography nicely highlights the challenge of getting rid of fictional bibliographies that lasted 50 years long in his case. On same line, eliminating inappropriate citations is a mandatory but difficult-to-achieve task for journals and citation platforms. This difficulty is highlighted by the fact that published studies that have been retracted due to scientific misconduct and other serious issues indeed frequently continue to be cited by researchers, despite being not valid and hence practically not existent anymore. Journals should face and manage this challenge. One solution might be the systematic linkage of retracted studies to citation platforms and journal editorial managers where the index reference/s, when cited, can then be easily identified and flagged during journal’s initial screen for duplicate publication, plagiarism, etc.. Another easier supportive tool is to make the reference crosscheck results visible not only to authors but also to reviewers and editors. Critical verification of non-validated references needs to be regular part of the peer review process. Finally, the probably increasing submission rates of AI-supported biomedical manuscripts necessitates declaration of using such tools. As the full spectrum of limits, risks and potentials inherent to these dynamically evolving innovative tools has yet to emerge, caution is mandatory, particularly among enthusiastic young researchers and scientists [7].
